# A Novel Strategy for Biomass Upgrade: Cascade Approach to the Synthesis of Useful Compounds via C-C Bond Formation Using Biomass-Derived Sugars as Carbon Nucleophiles

**DOI:** 10.3390/molecules21070937

**Published:** 2016-07-20

**Authors:** Sho Yamaguchi, Toshihide Baba

**Affiliations:** Department of Chemical Science and Engineering, School of Materials and Chemical Technology, Tokyo Institute of Technology, 4259-G1-14 Nagatsuta-cho, Midori-ku, Yokohama, Kanagawa 226-8502, Japan; baba.t.ab@m.tietch.ac.jp

**Keywords:** cascade reaction, biomass conversion, carbohydrate, Sn catalyst, aldol reaction

## Abstract

Due to the depletion of fossil fuels, biomass-derived sugars have attracted increasing attention in recent years as an alternative carbon source. Although significant advances have been reported in the development of catalysts for the conversion of carbohydrates into key chemicals (e.g., degradation approaches based on the dehydration of hydroxyl groups or cleavage of C-C bonds via retro-aldol reactions), only a limited range of products can be obtained through such processes. Thus, the development of a novel and efficient strategy targeted towards the preparation of a range of compounds from biomass-derived sugars is required. We herein describe the highly-selective cascade syntheses of a range of useful compounds using biomass-derived sugars as carbon nucleophiles. We focus on the upgrade of C2 and C3 oxygenates generated from glucose to yield useful compounds via C-C bond formation. The establishment of this novel synthetic methodology to generate valuable chemical products from monosaccharides and their decomposed oxygenated materials renders carbohydrates a potential alternative carbon resource to fossil fuels.

## 1. Introduction

**Scheme 1:**
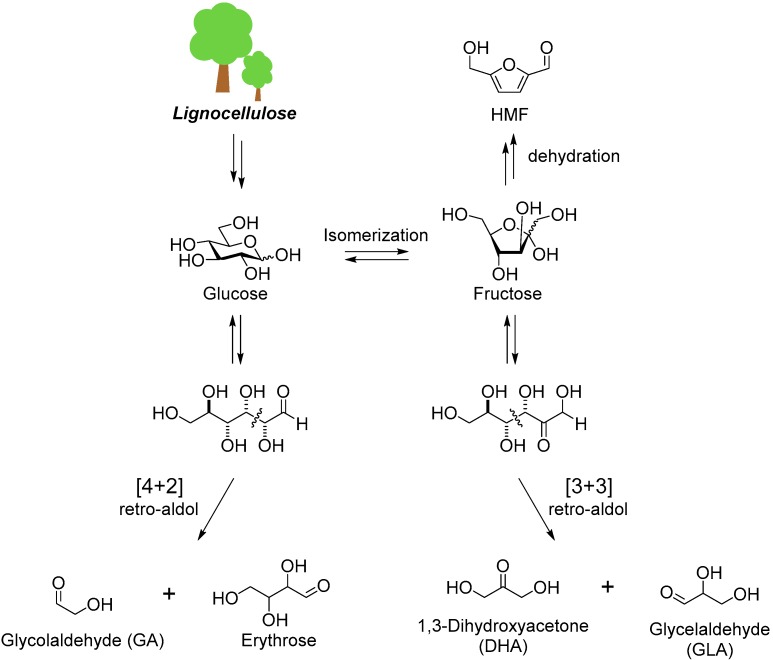
Transformation of lignocellulose-derived glucose into C2 (glycolaldehyde), C3 (1,3-dihydroxyacetone and glyceraldehyde), and C4 (erythrose) units via an isomerization and a retro-aldol reaction.

Currently, raw materials that can be converted into both chemical products and energy sources are elaborated from petroleum-based carbon resources. However, owing to the depletion of fossil fuels, the use of alternative renewable carbon resources is imperative in the field of green and sustainable chemistry. Recently, a number of studies have focused on carbohydrates derived from lignocellulosic materials (i.e., non-food resources) as abundant natural carbon resources. These sources represent the largest division of terrestrial biomass, and hence, various strategies to allow their efficient use as chemical feedstock are currently being developed, with the overall aim of supplementing and ultimately replacing fossil fuels [1,2,3,4,5,6]. In particular, the degradation of lignocellulose-derived cellulose into glucose is one of the most active topics in biomass conversion, and as such, the transformation of glucose into useful chemical products has attracted attention over the years. Indeed, significant advances have been reported in the development of catalysts for the conversion of monosaccharides into useful chemicals, including degradation approaches based on the dehydration of hydroxyl groups or the cleavage of C-C bonds via a retro-aldol reaction (Scheme 1) [7,8,9,10,11,12,13]. Under acidic conditions, the direct production of 5-hydroxymethyl furfural (HMF) proceeded under Brønsted acid catalysis [10]. Furthermore, glucose was decomposed to give erythrose (C4) and glycolaldehyde (GA, C2) via a [4 + 2] retro-aldol reaction. In contrast, 1,3-dihydroxyacetone (DHA, C3) and glyceraldehyde (GLA, C3) were obtained via an isomerization of glucose to fructose followed by a [3 + 3] retro-aldol reaction.

**Scheme 2:**
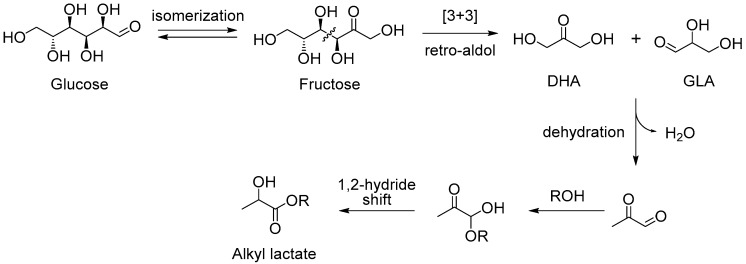
Conversion of monosaccharides to alkyl lactate via a retro-aldol reaction, dehydration, and 1,2-hydride shift.

As shown above, glucose can be theoretically converted into GA and erythrose via a [4 + 2] retro-aldol reaction; however, isomerization to fructose followed by a [3 + 3] retro-aldol reaction is thermodynamically favored over the glucose reaction. In 2010, Holm et al. reported a one-pot transformation of mono- and disaccharides into alkyl lactate via carbon-carbon bond cleavage, dehydration, and a 1,2-hydride shift (Scheme 2) [9]. These alkyl lactates are useful chemicals, as they can be employed as renewable solvents or as building blocks in polyester synthesis. For example, Lewis acidic homogeneous and heterogeneous Sn catalysts catalyze the conversion of mono- and disaccharides to methyl lactate in methanol solution. In particular, Sn-Beta zeolite yields the optimal performances in the conversion of glucose into methyl lactate. Beta type zeolites are composed of a three-dimensional (3D) 12-membered ring pore structures (6.6 Å × 7.6 Å), which allow the larger carbohydrates to spread through the zeolite pore. The reaction pathway in the acid-catalyzed conversion is highly sensitive to the type of acid employed. Brønsted acids catalyze monosaccharide dehydration, leading primarily to the formation of HMF and its decomposition products, while Lewis acidic catalysts lead to retro-aldol reactions of the monosaccharides, and subsequent transformation to their lactic acid derivatives. Therefore, to achieve high selectivity in the Lewis acid-catalyzed pathway, it is important to reduce the catalytic effect of Brønsted acids.

**Scheme 3:**
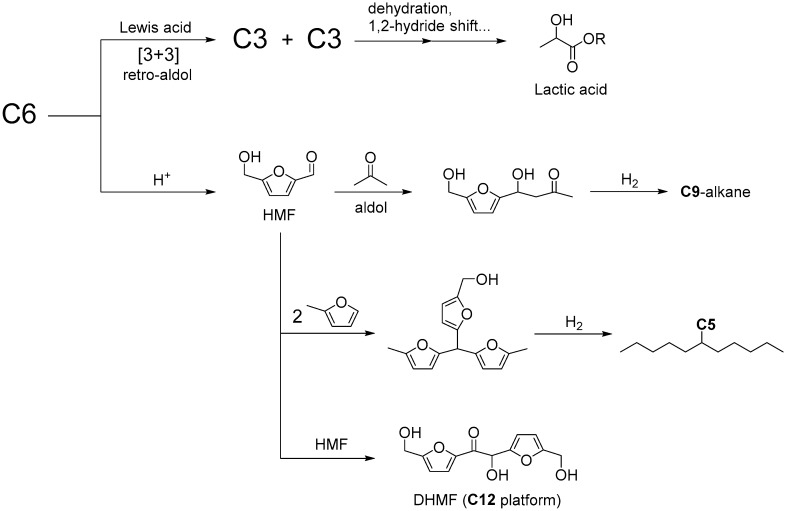
Conventional degradation methods of C6 units to yield a range of products.

**Scheme 4:**
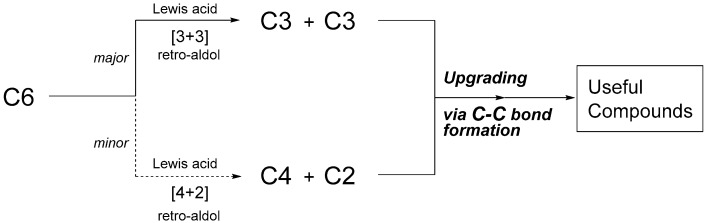
Upgrade of C2 and C3 oxygenates (generated from C6 units) via C-C bond formation.

The conventional degradation methods of the C6 unit into a range of compounds are shown in Scheme 3. In the case of a retro-aldol reaction, alkyl lactates are obtained as outlined in Scheme 2. In contrast, following the formation of HMF via sequential dehydration, further upgrade to various compounds takes place following aldol condensations with ketones [14], methylfuran [15], or HMF [16]. Although C-C bond cleavage via a retro-aldol and sequential hydroxyl group dehydration proceeds smoothly, a limited range of products is obtained through such a degradation process. Therefore, the development of a novel and efficient strategy targeted to the preparation of a wide range of compounds from biomass-derived sugars is required. We herein describe the highly selective cascade syntheses of a range of useful compounds using biomass-derived sugars as carbon nucleophiles. We focus on the upgrade of C2 and C3 oxygenates generated from glucose to yield useful compounds through C-C bond formation reactions (Scheme 4). The establishment of a novel synthetic methodology to generate valuable chemical products from monosaccharides and their decomposed oxygenated materials adds value to carbohydrates, rendering them suitable as alternative carbon resources.

## 2. Cascade Synthesis Using Biomass-Derived Oxygenates as Carbon Nucleophiles

**Scheme 5:**
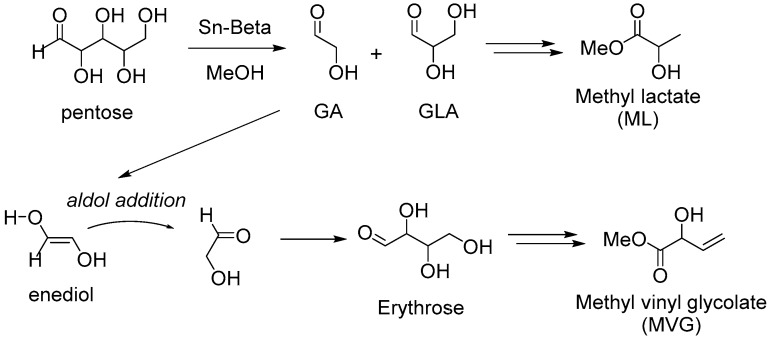
Sn-Catalyzed formation of GA in methanol and subsequent conversion of GA and its enediol (GLA) into vinyl glycolate esters.

In 2012, Holm et al. reported the conversion of various sugars into alkyl lactate using heterogeneous Sn-Beta zeolite [11]. Pentoses are converted to methyl lactate in slightly lower yields than those obtained for hexoses (Table 1, entries 1,2 vs. entries 3–7), which is in accordance with a reaction pathway involving the retro-aldol condensation of these sugars to form a triose and GA for the pentoses, and two trioses for the hexoses. When reacting GA (formally a C2-sugar) in the presence of a Sn catalyst, aldol condensation takes place, leading to the formation of methyl lactate, methyl vinyl glycolate, and methyl 4-methoxy-2-hydroxybutanoate (Table 2, entry 8 and Scheme 5).

**Scheme 6:**
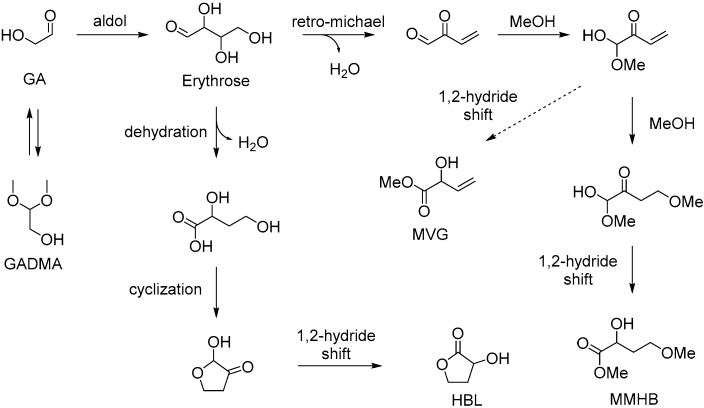
Cascade reaction of glycolaldehyde (GA) to give glycolaldehyde dimethyl acetal (GADMA) and α-hydroxy acids (methyl vinyl glycolate and methyl 4-methoxy-2-hydroxybutanoate) in alcoholic solution, and α-hydroxy-γ-butyrolactone (HBL) in non-alcoholic solvents.

Inspired by the above coupling with glycolaldehydes, in 2013, Sels et al. reported a catalytic route toward a series of recently discovered four-carbon α-hydroxy acids and their esters from the accessible and renewable glycolaldehyde (GA), which is prepared via the [4 + 2] retro-aldol reaction of glucose [17,18,19]. They reported the use of GA as both an aldol donor and an aldol acceptor, with an intermolecular aldol reaction followed by a sequential retro-Michael reaction, dehydration, and 1,2-hydride shift affording methyl-4-methoxy-2-hydroxybutanoate (MMHB). Indeed, Sn halides are unique in converting the small glycolaldehyde molecule into MMHB in methanol, with Brønsted acids and other Lewis acid catalysts yielding mainly glycolaldehyde dimethyl acetal (GADMA) (Table 2). When this reaction was performed in non-alcoholic solvents, such as acetonitrile, an intramolecular cyclization proceeded preferentially, to give a 5-membered lactone, namely α-hydroxy-γ-butyrolactone (HBL) (Scheme 6).

As mentioned in the Introduction, the balance between the Lewis and Brønsted acidities derived from the Sn halide salts is crucial in determining product selectivity. In the Figure 4 of ref [18], the influence of Brønsted:Lewis acid ratio on the overall reaction rate was also investigated. The highest rate of formation of the desired product was observed with a H^+^/Sn ratio of 3, and was independent of the oxidation state of the Sn salt. These results showed that the H^+^/Sn ratio was closely related to the rapid hydrolysis of acetals or the dehydration.

**Scheme 7:**
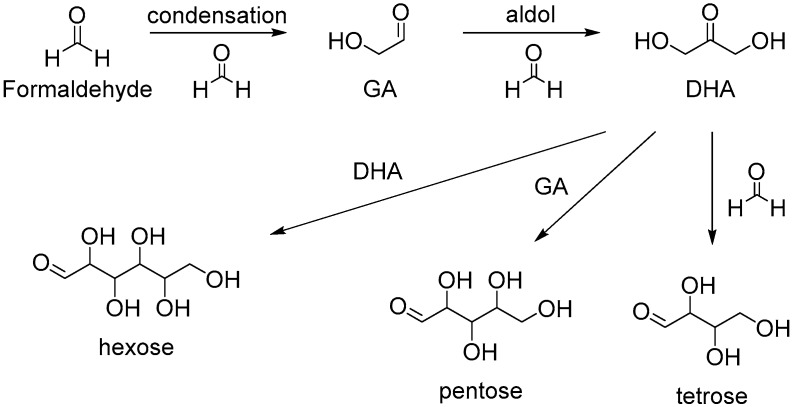
Preparation of unprotected sugar molecules based on the formose reaction.

The above-mentioned reactions based on an aldol reaction between the biomass-derived oxygenates (e.g., glycolaldehyde (GA)) are comparable to the formose reaction, in which sugars are prepared from formaldehyde and a sequential aldol condensation (Scheme 7) [20,21,22,23,24,25]. Although application of the formose reaction has previously been confined to the selective synthesis of unprotected sugars, an intermolecular coupling reaction between GA (C2) and oxygenates of different carbon numbers will increase the number of potential products.

## 3. Cascade Synthesis of Useful Four-Carbon Products via an Intermolecular Aldol Reaction between Biomass-Derived Triose Sugars and Formaldehyde

**Scheme 8:**
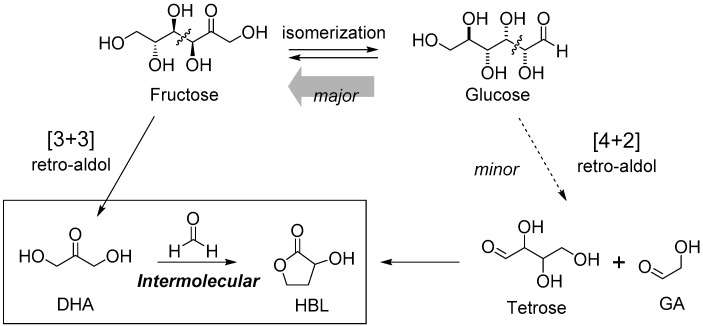
Synthetic route to the useful four-carbon product, α-hydroxy-γ-butyrolactone (HBL) via an intermolecular aldol reaction between 1,3-dihydroxyacetone (DHA) and formaldehyde.

A previously discussed, the isomerization of glucose and the [3 + 3] retro-aldol reaction of fructose are thermodynamically more favorable than the [4 + 2] retro-aldol reaction. Thus, to efficiently obtain chemical products from biomass-derived sugars, the development of a novel synthetic strategy employing C3 units is desired. Our group therefore focused on a novel route to the cascade synthesis of 5-membered lactones [26,27], which can be utilized not only as fine chemicals, but also as raw materials [28,29,30,31,32,33]. This route employed a biomass-derived triose sugar (1,3-dihydroxyacetone, DHA) as the carbon nucleophile and aldehydes as the electrophiles (Scheme 8). Indeed, if such an efficient route to useful compounds from biomass-derived sugars can be established, the demand for carbohydrates as alternative carbon resources will increase.

### 3.1. Tin-Catalyzed Conversion of the Biomass-Derived Triose Sugar 1,3-Dihydroxyacetone (DHA) to α-Hydroxy-γ-butyrolactone (HBL) in the Presence of Formaldehyde

**Scheme 9:**
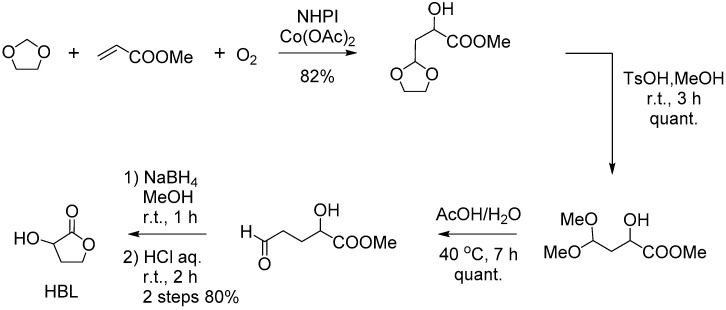
A novel route to α-hydroxy-γ-butyrolactone (HBL) via a three-component radical coupling of 1,3-dioxoranes, acrylates, and molecular oxygen.

As mentioned above, 5-membered lactones are useful not only as fine chemicals but also as raw materials. In particular, 5-membered lactones possessing a hydroxyl group in the α-position are also attractive synthetic targets, with the majority of chemical [34,35,36,37,38,39,40,41,42,43,44,45] or enzymatic [46,47,48] syntheses being based on multistep processes. For example, Kagayama et al. reported the multi-step synthesis of α-hydroxy-γ-butyrolactone (HBL), one of the simplest 5-membered lactones, from 1,3-dioxorane and methyl acrylate, which are relatively cheap commercially available compounds, in the presence of an *N*-hydroxyphthalimide (NHPI) catalyst (Scheme 9) [37]. The addition of 1,3-dioxarane to methyl acrylate under oxygen in the presence of NHPI followed by treatment with acids and subsequent NaBH_4_ reduction of the resulting adduct afforded HBL in good yield. This method therefore provides an efficient approach to HBL, which is difficult to synthesize by conventional means. However, the development of a more efficient and a widely applicable approach from cheap and easily available starting materials would be extremely welcomed in terms of green and sustainable chemistry.

The synthetic target of this process is HBL, which was obtained as a byproduct in the reaction of GA in non-alcoholic solvents, as shown in Scheme 6. However, in the novel route, the reaction between DHA and formaldehyde was examined, with the catalysts used and yields of the identified products (i.e., HBL, LA and VG) being given in Table 3 [26,27]. A number of metal chloride catalysts previously reported for DHA activation were screened, including AlCl_3_, ZrCl_4_, and TiCl_4_, but these catalysts had no effect on the reaction. However, when SnCl_4_·5H_2_O was employed, HBL was obtained in 42% yield, and LA and VG were generated as by-products in 12% and 6% yield, respectively (entry 2). In addition, the combination of anhydrous SnCl_4_ with a small amount of water increased the reaction efficiency, giving HBL in 63% yield (entry 8). Homogeneous Sn halides were therefore confirmed to display specific catalytic activity for the desired coupling reaction between DHA and formaldehyde.

#### 3.1.1. Cascade Reaction Mechanism

**Scheme 10:**
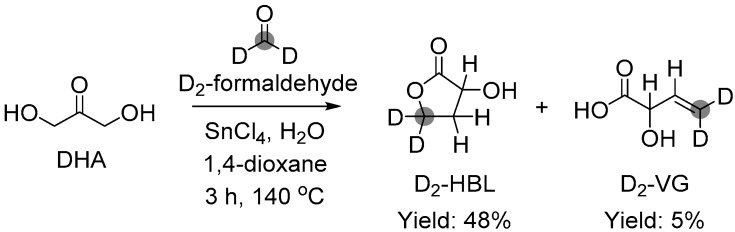
Reaction of DHA and formaldehyde-*d_2_*.

To confirm that the reaction between DHA and formaldehyde to yield HBL proceeded via an intermolecular reaction, an incorporation experiment using formaldehyde-*d*_2_ was conducted. The reaction between DHA and formaldehyde-*d_2_* using SnCl_4_ in the presence of a small amount of water generated HBL-*d*_2_ in 48% yield, where the deuterium atoms were incorporated adjacent to the ether oxygen atom (Scheme 10). Product formation in the batch experiments was monitored as a function of reaction time. After 60 min, the DHA supply had been almost completely exhausted, and the presence of the pyruvic aldehyde (PA) intermediate could be detected. DHA therefore appeared to firstly tautomerize rapidly to GLA, with PA forming as an intermediate, as implied by its higher concentrations in the early stages of the reaction. Erythrulose was not detected, thus supporting the hypothesis of a PA intermediate.

**Scheme 11:**
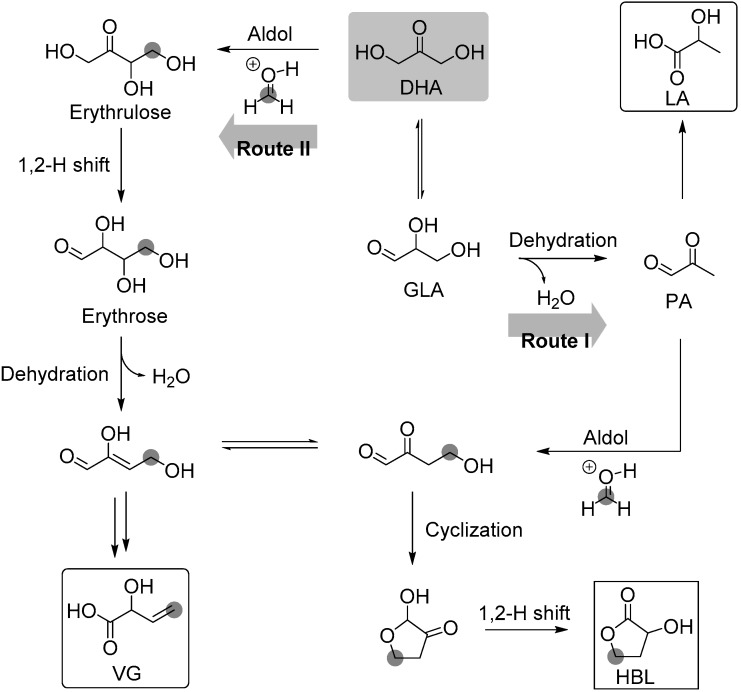
Proposed reaction pathways to HBL, VG, and LA.

Based on the above results, two plausible reaction mechanisms were proposed, as outlined in Scheme 11 [27]. An initial tautomerization between DHA and GLA proceeds because elimination of the hydroxyl group from the β-position of GLA appears more favorable than elimination from DHA due to the arrangement of the atomic orbitals. Sn can then coordinate with multiple –OH or =O moieties on PA due to its strong Lewis acid character, resulting in the formation of the activated complex and subsequent aldol reaction with formaldehyde to afford the diketone intermediate. The resulting molecule is likely prone to intramolecular esterification in the presence of HCl generated from the Sn halides, thus leading to the formation of HBL (Route I). In contrast, an alternative route to HBL involves the aldol reaction between DHA and formaldehyde (Route II). Following initial aldol coupling and tautomerization, the aldehyde appears prone to elimination of the hydroxyl group in the β-position of the unsaturated carbon. Dehydration at the C3 position of erythrose provides the enone intermediate, which can lead to the formation of either VG or HBL. To validate these proposed reaction pathways, an experiment with erythrulose was carried out, affording HBL in only 9% yield and VG in 31% yield. These results confirm that Route I was the main path followed in the proposed mechanism.

However, a PA induction period was observed, with the rate of LA formation being higher than that of both HBL and VG. Furthermore, PA was the only reaction intermediate observed, suggesting that the rate-determining step of this reaction could be an aldol reaction between the enol form of PA and formaldehyde.

#### 3.1.2. Specific Catalysis by Sn Halides in the Synthesis of HBL

**Scheme 12:**
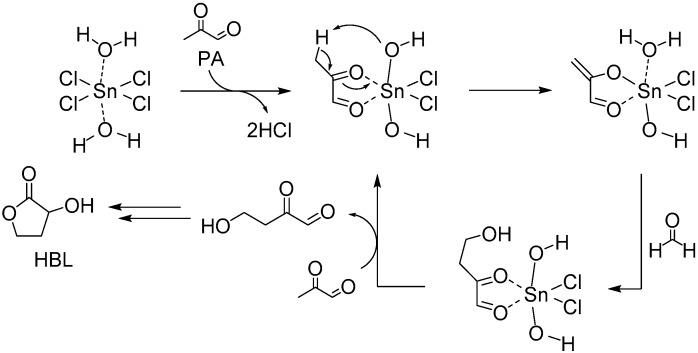
Proposed route to HBL from PA in the presence of a SnCl_4_(H_2_O)_2_ catalyst.

As the balance between Lewis and Brønsted acidity influences product yields, the interaction between the Lewis acidic (Sn) and Brønsted acidic (HCl) species derived from the Sn chloride salts in the reaction medium is crucial to the overall rate of this cascade reaction. The balance between Lewis and Brønsted acidity is therefore key to understanding this cascade reaction, allowing optimization of higher yields and selectivities. Investigation into the influence of the HCl/Sn ratio on product yields confirmed that the optimal H^+^/Sn ratio for this system was 4 for both SnCl_4_·5H_2_O or SnCl_2_·2H_2_O [27]. Furthermore, based on ^119^Sn NMR studies, a solution of SnCl_4_·5H_2_O in CD_3_CN-*d_3_* generates the 6-coordinate complex SnCl_4_(H_2_O)_2_, which is the active catalytic species in this system. Based on these results and the rate-determining step identified in Scheme 11 (i.e., an intermolecular aldol reaction), following ligand exchange between the active SnCl_4_(H_2_O)_2_ species and PA, the reaction is accelerated by an aldol reaction and subsequent elimination of the diketone intermediate (Scheme 12). This therefore accounts for the observed effects upon the addition of water, as identified in Table 3, entry 9.

Prior to this study, the synthesis of HBL was particularly challenging, and so this novel coupling between DHA and formaldehyde could aid in establishing wood biomass as a source functional compounds. Furthermore, the results presented herein could lead to the development of more sustainable heterogeneous tin catalysts, such as zeolites or silica containing immobilized Sn, which have the potential for Sn regeneration.

### 3.2. A Sugar-Accelerated Tin-Catalyzed Cascade Synthesis of α-Hydroxy-γ-butyrolactone from Formaldehyde

Formaldehyde, which is produced industrially by the catalytic oxidation of methanol, is a naturally-occurring organic compound and is an important precursor to many other materials and chemical compounds. In the presence of basic catalysts, formaldehyde undergoes the formose reaction to produce carbohydrates, as previously outlined in Scheme 7 [20,21,22,23,24,25]. Butlerov proposed a mechanism for this reaction, where two formaldehyde molecules condense to form GA, which subsequently reacts via an aldol reaction with another equivalent of formaldehyde to afford GLA. The aldose-ketose isomerization of GLA then yields DHA, which can react with formaldehyde, GA, and GLA; subsequent isomerization results in the formation of tetrose, pentose, and hexose, respectively. To date, successful examples of the formose reaction, developed exclusively for the selective syntheses of unprotected sugars, have been carried out under either basic or mineral conditions. However, to the best of our knowledge, with the exception of saccharide synthesis, application of the formose reaction to produce other important chemicals has not been reported. This is likely due to issues regarding control of the formose reaction, i.e., the coupling frequency of formaldehyde and handling of the product mixtures.

In 2015, our group reported a novel tin chloride-catalyzed cascade synthesis of HBL from formaldehyde alone. We observed that the addition of mono- and disaccharides had an accelerating effect on HBL formation, as outlined in Table 4 [49,50]. When glucose was used as an accelerator for this reaction, HBL yields increased dramatically (entries 1 and 2 vs. entries 3 and 4). The results suggested that the presence of adjacent carbonyl and hydroxyl groups, i.e., an α-hydroxy carbonyl moiety, is essential in achieving this accelerating effect. Indeed, the cascade conversion of formaldehyde into HBL is accelerated by the addition of other compounds bearing a terminal α-hydroxy carbonyl moiety, such as hydroxyacetone and hydroxyacetphenone (entries 5, 8 and 9 vs. entries 6, 7, 10 and 11).

**Scheme 13:**
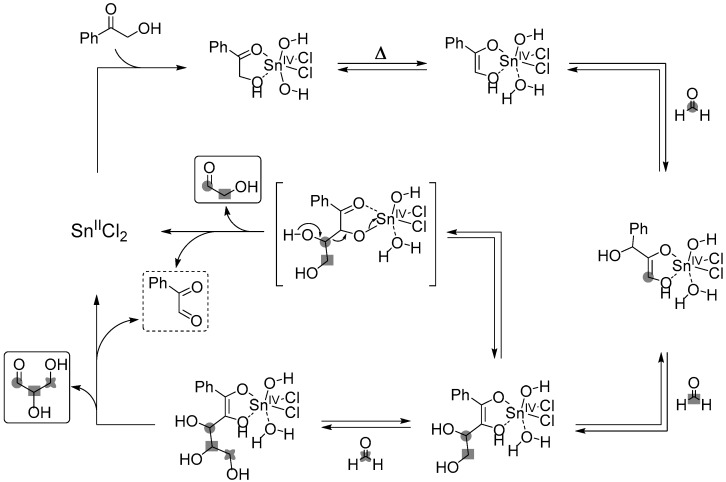
Proposed route to GA and GLA (intermediates in the synthesis of HBL) from four molecules of formaldehyde in the presence of a tin catalyst complex and an accelerator.

^119^Sn NMR spectroscopy and X-ray absorption fine structure (XAFS) measurements clarified the oxidation states of the various tin complexes, allowing a potential mechanism for HBL formation from four formaldehyde molecules to be determined (Scheme 13). Upon heating, the tetravalent tin complex generated from SnCl_2_·2H_2_O and the accelerator is initially converted into a complex bearing an ene-diol ligand. A subsequent aldol condensation with formaldehyde, followed by a 1,2-hydride shift and C-C bond formation takes place. Phenylglyoxal (PG) and either GA or GLA are then produced via the retro-aldol reaction of a ligand, reducing the tetravalent tin complex to a divalent complex. Based on the proposed mechanism, the use of substrates bearing a non-terminal α-hydroxy carbonyl moiety (e.g., acetoin or benzoin), does not lead to an accelerating effect, as the α-hydroxy carbonyl moiety donates the hydrogen atom of the α-carbon to the aldol reaction with formaldehyde, thus terminating the reaction.

In contrast, Matsumoto et al. reported the selective synthesis of triose sugars from formaldehyde by the use of 3-ethylbenzothiazolium bromide as a catalyst in the presence of base [51]. Interestingly, only C3 products were obtained as major products. These results allow an effective comparison between acidic and basic catalysis, indicating that the use of acid catalysts may lead to the formation of compounds containing more than 3 carbon atoms.

### 3.3. Application of Heterogeneous Catalysts in the Intermolecular Aldol Reaction between 1,3-Dihydroxyacetone and Formaldehyde

**Figure 1:**
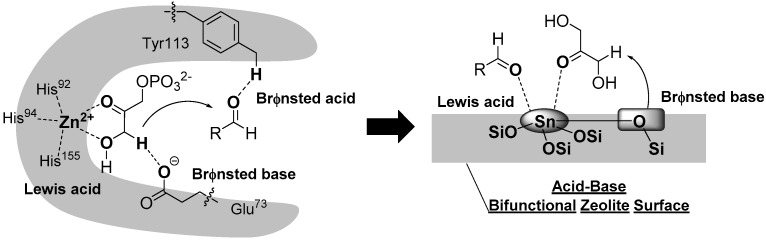
Acid-base cooperative catalysis inspired by a three-dimensional enzyme architecture.

Up to this point, we have described the cascade synthesis of 5-membered lactones in the presence of homogeneous tin catalysts. However, from an industrial viewpoint, the use of renewable heterogeneous catalysts and a decrease in catalyst toxicity are desirable. In 2015, Román-Leshkov et al. reported the application of heterogeneous Sn catalysts in the catalytic C-C coupling between DHA and formaldehyde to form HBL [52]. This reaction proceeds on the surface of the solid catalysts inspired by a three-dimensional enzyme architecture (Figure 1) [53,54]. The enzyme, a class II aldolase, catalyzes a direct asymmetric aldol reaction between dihydroxyacetone phosphate (DHAP) and various aldehydes based on cooperative activation [55,56,57,58,59,60,61,62,63]. Inspired by this intriguing mechanism, they related an enzymatic function to acid-base cooperative catalysis on the zeolite surface, where the Lewis acidic framework Sn atom polarizes the carbonyl group of DHA, and an α-proton can be removed by the basic oxygen atom connected to Sn. This sequence (i.e., soft enolization [64,65,66,67]) results in the successful coupling reaction between DHA and formaldehyde.

**Scheme 14:**
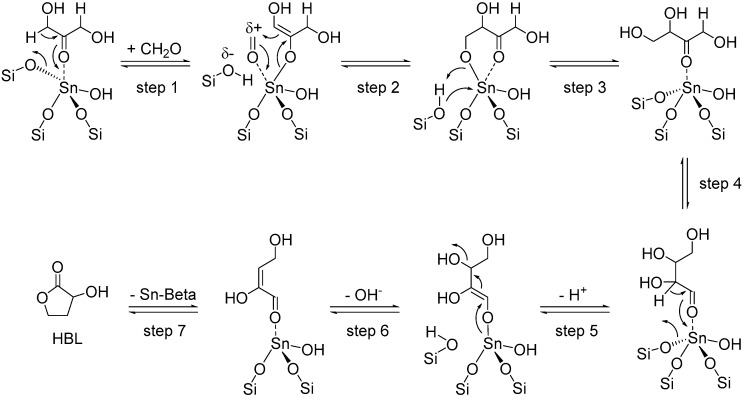
Cooperative catalysis of a Lewis acid Sn center and a Brønsted base oxygen atom in the Si-O-Sn framework.

Table 5 shows the catalyst screening results for this reaction, with the Sn-Beta zeolite giving the highest selectivity, generating HBL in 60% yield and 98% conversion after 3 h [52]. For the reactions performed in batch mode, Sn-Beta zeolite and SnCl_4_ afforded 68 and 70% yield, respectively, with almost complete conversion. The critical aldol condensation step is promoted by the cooperative catalysis of a Lewis acid Sn center and a Brønsted base oxygen atom in the Si-O-Sn framework. This proposed mechanism is outlined in Scheme 14. Following coordination of the carbonyl oxygen atom of DHA to the Lewis acidic Sn center, the α-proton can be removed by the weakly basic oxygen atom in the Si-O-Sn framework (step 1). The electrophilicity of the carbonyl moiety is increased by the coordination of formaldehyde, and C-C bond formation and a proton transfer from the silanol moiety proceed (steps 2 and 3). A 1,2-hydride shift and dehydration then yield an enone intermediate (steps 4–6). Finally, an intramolecular cyclization, 1,2-hydride shift, and elimination of the substrate from the catalyst surface provide the desired product (step 7). This proposed reaction pathway differs from that postulated by our group for Sn halides, which involves the tautomerization and dehydration of DHA to give PA. However, in both cases, the formation of Sn enolates is proposed through the deprotonation of a C-H bond in the α-position to the carbonyl group of either DHA or PA.

## 4. Cascade Synthesis of 5-Membered Lactones Using Biomass-Derived Sugars and a Range of Aldehydes

**Scheme 15:**
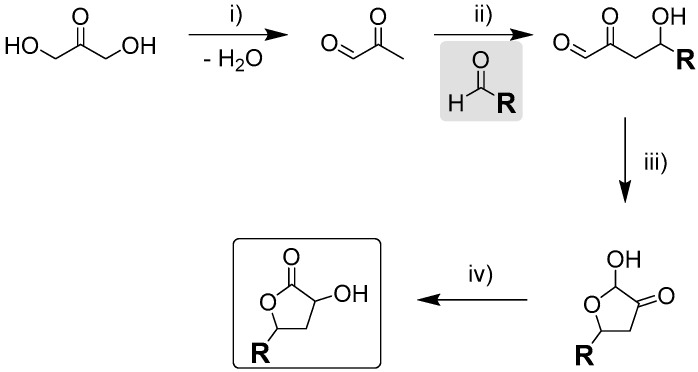
Cascade reaction of DHA and aldehydes via sequential (**i**) isomerization and dehydration of DHA; (**ii**) aldol reaction; (**iii**) cyclization; and (**iv**) 1,2-hydride shift.

The use of aldehydes instead of formaldehyde can yield a wide range of products in the multistep HBL preparation process. Indeed, our group reported the cascade synthesis of 5-membered lactones using a combination of DHA and various aldehydes [68] (Scheme 15). Such a synthetic route is particularly advantageous, as 5-membered lactones can be utilized not only as fine chemicals but also as raw materials. The majority of previous reports on the chemical [34,35,36,37,38,39,40,41,42,43,44,45] or enzymatic [46,47,48] syntheses of 5-membered lactones are based on multistep processes, and so the development of a more efficient and widely applicable approach from inexpensive and easily available starting materials is desirable.

**Scheme 16:**
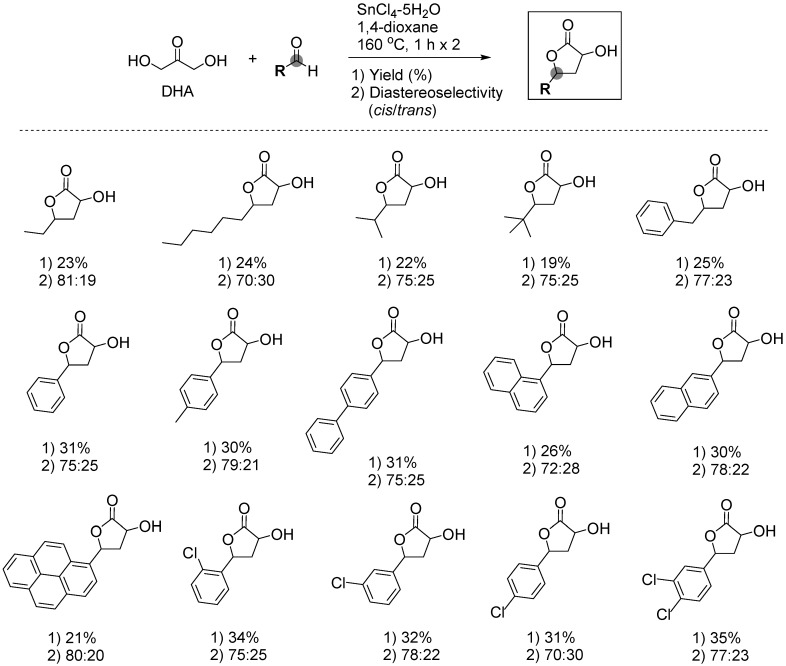
Cascade synthesis of a range of useful compounds using a biomass-derived triose sugar (DHA) as the carbon nucleophile and various aldehydes as electrophiles.

A wide range of 5-membered lactones, including both important chemicals and bioactive compounds were obtained, as outlined in Scheme 16. Furthermore, the reaction proceeded stereoselectively, with the cis product being obtained as the major product. Stability tests of the cis/trans products along with DFT calculations showed that the observed diastereoselectivity was controlled by the thermodynamic stabilities of the products [69]. In contrast, the use of aldehydes gave lower yields, likely due to their increased steric hindrance and decreased electrophilicity.

## 5. Conclusions

In this review, we have highlighted the potential of biomass-derived sugars for the cascade synthesis of valuable chemical products. Although significant advances have been reported in the development of catalysts for the conversion of carbohydrates into useful compounds, such as the degradation of hydroxyl groups or C-C bond cleavage via a retro-aldol reaction, only a limited range of products can be obtained through these processes. To overcome such challenges, we identified novel synthetic methodologies for the preparation of such compounds through the use of biomass-derived sugars as carbon nucleophiles.

A C2 glycolaldehyde (GA) obtained by a [4 + 2] retro-aldol reaction of glucose can be converted into erythrose (C4) via an intermolecular aldol reaction followed by dehydration and a 1,2-hydride shift, yielding vinyl glycolate, which is of great appeal for use as a renewable solvent and as a building block for polyester synthesis. Furthermore, 1,3-dihydroxyacetone (DHA), prepared by a sequential isomerization of glucose and a retro-aldol reaction of fructose, can be converted into 5-membered lactones via an intermolecular aldol reaction with formaldehyde (C1). The use of solid catalysts as alternatives to homogeneous Sn catalysts led to a green and sustainable process. Indeed, the behavior of Sn-Beta zeolites was comparable to that of an enzyme. The achievements described herein are therefore likely to lead to novel synthetic strategies for the generation of a wide range of valuable compounds from the combination of biomass-derived triose sugars and electrophiles.

## Figures and Tables

**Table 1:**
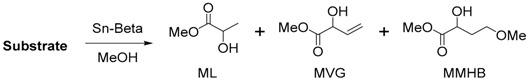
Conversion of various sugars in methanol using Sn-Beta ^1^.

Entry	Substrate	Conversion (%)	Yield (%)		
			ML	MVG	MMHB
1	Xylose	98	42	7	<2
2	Ribose	96	38	8	<2
3	Glucose	98	51	10	<2
4	Fructose	98	54	11	<2
5	Mannose	96	47	9	<2
6	Galactose	95	45	5	<2
7	Sucrose	92	57	5	0
8	Glycolaldehyde	-	16	27	6

^1^ Yields of methyl lactate (ML), methyl vinyl glycolate (MVG) and methyl 4-methoxy-2-hydroxybutanoate (MMHB) for the conversion of various sugars catalyzed by Sn-Beta zeolite. Reaction conditions: substrate (300 mg), methanol (10 g), Sn-Beta (100 mg), 160 °C, 16 h.

**Table 2:**
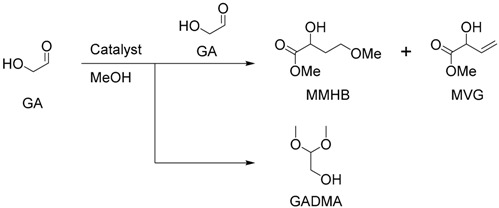
Conversion of glycolaldehyde into α-hydroxy acid esters and GADMA in CH_3_OH ^1^.

Entry	Catalyst	Time (h)	Yield (%)		
			GADMA	MMHB	MVG
1	none	26	47	0	0
2	HCl	22	56	0	0
3	NaOH	20	0	0	0
4	SnCl_4_·5H_2_O	1	54	10	<1
5	SnCl_4_·5H_2_O	20	3	58	3
6	SnCl_2_·2H_2_O	1	51	14	<1
7	SnCl_2_·2H_2_O	21	1	55	4
8	AlCl_3_·6H_2_O	20	47	7	0
9	CrCl_3_·6H_2_O	20	71	<1	0
10 ^2^	SnCl_4_·5H_2_O	1	-	50	2

^1^ Reactions carried out at 90 °C using 1.25 M GA in MeOH, 5 mol % of catalyst (20 mol % for HCl, 10 mol % for NaOH). Yields were derived from GC; ^2^ Reaction using 0.625 M erythrose in CH_3_OH, with 10 mol % catalyst.

**Table 3:**
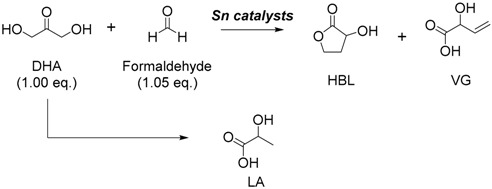
Conversion of DHA and formaldehyde into HBL, VG, and LA in the presence of Sn catalysts ^1^.

Entry	Catalysts	Conversion of DHA (%)	Yield (%)		
			HBL	VG	LA
1	HCl	>99	0	0	21
2	SnCl_4_·5H_2_O	>99	42	6	12
3	SnCl_2_·2H_2_O	>99	24	3	13
4 ^2^	SnCl_2_·2H_2_O	>99	37	4	16
5	*n*Bu_3_SnCl	>99	25	3	12
6	SnO_2_	95	0	0	<1
7 ^3^	SnCl_4_	>99	40	4	17
8	SnCl_4_	>99	63	8	20
9 ^4^	SnCl_4_	>99	70	5	5
10	AlCl_3_	>99	0	0	5
11	TiCl_4_	>99	0	0	2
12	ZrCl_4_	>99	0	0	18
13	Sn(OAc)_2_	>99	0	0	5
14	Sn(OTf)_2_	>99	0	0	36
15	none	79	0	0	4

^1^ Reaction conditions: DHA (1.25 mmol), paraformaldehyde (1.31 mmol), catalyst (0.171 mmol), 1,4-dioxane (4.0 mL), Ar, 3 h, 140 °C. Unless otherwise noted, the total amount of water in all entries was fixed at 0.86 mmol. Yields are based on DHA; ^2^ 4 M HCl in 1,4-dioxane (0.34 mmol) was added; ^3^ No additional water; ^4^ Excess paraformaldehyde (3.75 mmol) was employed.

**Table 4:**
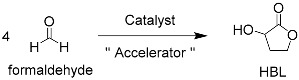
Screening of accelerators bearing an α-hydroxy carbonyl moiety ^1^.

Entry	Catalysts	Accelerator	HBL (mmol) ^2^	Yield of HBL (%) ^2^
1	SnCl_4_·5H_2_O	-	0.02	1
2	SnCl_2_·2H_2_O	-	0.17	11
3	SnCl_4_·5H_2_O	Glucose 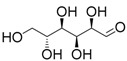	0.33	-
4	SnCl_2_·2H_2_O	Glucose	0.53 (0.41) ^3^	26
5		1,3-Dihydroxyacetone 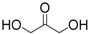	0.52 (0.39) ^3^	25
6		Sorbitol 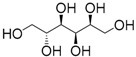	0.16	10
7		Glucosamine 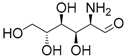	<0.01	<1
8		Hydroxyacetone 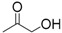	0.34	22
9		Hydroxyacetphenone 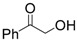	0.39	25
10		Acetoin 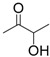	0.06	4
11		Benzoin 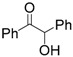	0.07	5

^1^ Reaction conditions: Accelerator (0.625 mmol), paraformaldehyde (6.25 mmol), 1,4-dioxane (4.0 mL), catalyst (0.043 mmol), naphthalene (20 mg), air atmosphere, 3 h, 160 °C; ^2^ HBL quantity was determined by ^1^H-NMR analysis; ^3^ Derived from formaldehyde.

**Table 5:**
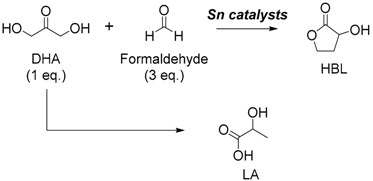
Results for the Lewis Acid catalyzed synthesis of HBL ^1^.

Entry	Catalysts	DHA Conversion (%)	Yield (%) ^2^	
			HBL	LA
1	Sn-Beta	98	60	9
2	Sn-Beta ^3^	98	68	8
3	Zr-Beta	99	3	2
4	Hf-Beta	99	7	3
5	Ti-Beta	96	6	3
6	Al-Beta	95	11	6
7	Sn-MCM-41	99	64	15
8	Sn-MFI	90	61	6
9	SnO_2_/Si-Beta	53	3	<1
10	none	12	<1	<1

^1^ Reaction conditions: DHA (2.4 mmol), paraformaldehyde (7.2 mmol), 1,4-dioxane (8.0 mL), 0.024 mmol metal in the added catalysts, 20 bar Ar at r.t., 160 °C, 3 h; ^2^ Determined by GC/FID and expressed relative to the initial molar amount of DHA; ^3^ Reaction performed in the presence of water (2.4 mmol).
